# A gene expression inflammatory signature specifically predicts multiple myeloma evolution and patients survival

**DOI:** 10.1038/bcj.2016.118

**Published:** 2016-12-16

**Authors:** C Botta, M T Di Martino, D Ciliberto, M Cucè, P Correale, M Rossi, P Tagliaferri, P Tassone

**Affiliations:** 1 Department of Experimental and Clinical Medicine, Magna Graecia University, Catanzaro, Italy; 2 Department of Oncology, Siena University, Siena, Italy

## Abstract

Multiple myeloma (MM) is closely dependent on cross-talk between malignant plasma cells and cellular components of the inflammatory/immunosuppressive bone marrow *milieu*, which promotes disease progression, drug resistance, neo-angiogenesis, bone destruction and immune-impairment. We investigated the relevance of inflammatory genes in predicting disease evolution and patient survival. A bioinformatics study by Ingenuity Pathway Analysis on gene expression profiling dataset of monoclonal gammopathy of undetermined significance, smoldering and symptomatic-MM, identified inflammatory and cytokine/chemokine pathways as the most progressively affected during disease evolution. We then selected 20 candidate genes involved in B-cell inflammation and we investigated their role in predicting clinical outcome, through univariate and multivariate analyses (log-rank test, logistic regression and Cox-regression model). We defined an 8-genes signature (IL8, IL10, IL17A, CCL3, CCL5, VEGFA, EBI3 and NOS2) identifying each condition (MGUS/smoldering/symptomatic-MM) with 84% accuracy. Moreover, six genes (IFNG, IL2, LTA, CCL2, VEGFA, CCL3) were found independently correlated with patients' survival. Patients whose MM cells expressed high levels of Th1 cytokines (IFNG/LTA/IL2/CCL2) and low levels of CCL3 and VEGFA, experienced the longest survival. On these six genes, we built a prognostic risk score that was validated in three additional independent datasets. In this study, we provide proof-of-concept that inflammation has a critical role in MM patient progression and survival. The inflammatory-gene prognostic signature validated in different datasets clearly indicates novel opportunities for personalized anti-MM treatment.

## Introduction

Multiple myeloma (MM) is one of the most common hematologic malignancies and is characterized by an uncontrolled clonal proliferation of malignant plasma cells (PCs) within the bone marrow (BM). MM is considered a multistep disease, as it progress from monoclonal gammopathy of undetermined significance (MGUS),^1^ that evolves in MM in about 1% of cases per year, often with the intermediate phase of smoldering MM (sMM).^2^ Although lacking the clinical features of symptomatic disease, both MGUS and sMM patients carry the same initial mutations and most of the chromosomal abnormalities of overt MM, suggesting that these events are necessary but not sufficient for disease progression.^3, 4^ The evolution from MGUS to sMM and finally to MM relies on further complex conditions that include genomic instability, epigenetic and microenvironmental signals.^2, 4, 5^ The interplay between MM cells and the BM microenvironment (BMM) is currently under active investigation, and different studies have pointed out its role in both disease pathogenesis and progression.^3, 6^ Indeed, MM cells grow and proliferate almost exclusively within the BM, where they produce an inflammatory/immunosuppressive *milieu*, which promotes disease progression, drug resistance, neo-angiogenesis, bone destruction and immune escape.^7, 8, 9^

Inflammation has been recently recognized as hallmark of cancer because of its role in cancer initiation and progression.^10^ Cytokines and chemokines produced in the tumor microenvironment by cancer or cancer-associated cells (such as immune infiltrating cells), have been reported to support cancer cell growth, and induce epigenetic changes and genomic instability.^11, 12, 13^

On these bases, we identified an inflammatory-gene signature able to discriminate the different phases of disease progression. Moreover, we investigated the prognostic relevance of inflammatory-gene expression in predicting MM patient survival by analyzing large annotated gene expression profiling (GEP) datasets.

## Material and methods

### Gene expression datasets

GEP data from five different datasets underwent our statistical analysis (datasets characteristics are reported in Supplementary Table 1): (1) GSE47552^(ref. 2)^ including GEP data from purified CD138+ cells from BM of five healthy donors, 20 MGUS, 33 sMM and 41 newly diagnosed MM patients; (2) GSE9782^(ref. 14)^ including GEP data from 264 pretreated patients enrolled in phase II and III bortezomib trials; (3) GSE24080^(ref. 15)^ including GEP data from 559 newly diagnosed MM treated with total therapy (TT) 2 or 3; (4) GSE57317^(ref. 16)^ including GEP data from 55 pretreated patients enrolled in TT6 phase II clinical trial; and (5) GSE2658 ^(ref. 17)^ including GEP data from 559 chemo-naive patients enrolled in TT2 and TT3 clinical trials. According to original studies, gene expression data from different datasets were normalized independently by using the microarray suite 5.0 (MAS5, Affymetrix, Santa Clara, CA, USA) algorithm (except for GSE47552, normalized with the robust multi-array analysis (RMA) algorithm). GEP data from GSE47552 dataset underwent fold-change (FC) analysis by using dChip software.^18^ The comparison analysis tool of Ingenuity Pathway Analysis (IPA) (Ingenuity System, Redwood city, CA, USA) was used to evaluate the main pathways modulated during disease progression from MGUS to sMM and to MM. To fulfill IPA requirements for this analysis, each condition (MGUS, sMM and MM) was compared to normal samples (that in this case worked as ‘normalizer') and then the three different FC analyses underwent a comparison study to investigate the main modulated canonical pathways. A fold-change >1.5 and a *P*-value <0.05 were used to include genes in the IPA analysis.

### Inflammatory model to discriminate between MGUS/sMM/MM

A shortlist of 20 candidate genes coding for cytokines/chemokines involved in inflammatory response has been derived by relevant literature, focusing on B lymphocytes (effector/regulatory) or healthy/malignant PCs: IL2, IL6, IL8, IL10, IL12A, IL15, IL17A, EBI3 (IL35), CCL2 (MCP1), CCL3 (MIP1a), CCL5 (RANTES), CSF2 (GM-CSF), VEGFA, TNF, NOS2 (iNOS), IFNG, TNFSF11 (RANK-ligand), LTA (Lymphotoxin A/TNF-b), LTB, TGFB1;^19, 20, 21, 22, 23, 24, 25, 26, 27, 28^ (Supplementary Table 2). The expression level of these genes was retrieved from each dataset and used for further analyses. When multiple probes were found to map to the same gene, the one with the highest values was used. All genes were evaluated for their capability to discriminate between MGUS/sMM/MM through univariate analysis by using a non-parametric Kruskal–Wallis one-sided ANOVA. Subsequently, all significant variables (*P*<0.05), underwent a multinomial logistic regression model, where the variables significantly associated with the disease status were identified by using a backward Wald approach. The receiving operating characteristic (ROC) curve analysis was used to evaluate the performance of the model for disease status prediction in terms of sensitivity and specificity.

### Construction of an inflammatory prognostic score

We used the GSE9782 dataset (excluding patients treated with dexamethasone alone) to build an inflammatory prognostic score, whereas GSE24080, GSE57317 and GSE2658 datasets were used to validate the model. Each of the 20 selected genes was divided into three categories according to their expression (low/mid/high expression, using the 33th and 66th percentile as cut-offs) and was evaluated in a univariate analysis for its association with patients' overall survival (OS) by using the log-rank test. Subsequently, only variables associated with survival (*P*-value selected at <0.1) were entered into a Cox proportional hazard regression model. The variables resulted independently associated with survival (*P*<0.05) were further used to build a prognostic score and patients were divided into three prognostic groups (PGs) (high risk/mid risk/low risk). ROC curve and log-rank test were used to evaluate the prognostic performance of the model.

### Statistical analysis

Parametric and non-parametric tests were used to compare means between groups, according to Gaussian or not-Gaussian distribution of the variable evaluated. Survival curves were built through the Kaplan–Meier method and differences between groups evaluated through the log-rank test. Multivariate analysis was performed through the logistic regression model for classification or Cox-regression model for survival analysis. All statistical analyses were performed though SPSS 20.0 and Graphpad PRISM 6 statistic packages. This work follows reporting recommendations for tumor marker prognostic studies (REMARK).^29^

## Results

### An 8-genes signature correctly differentiates between MGUS, sMM and MM

**Figure 1:**
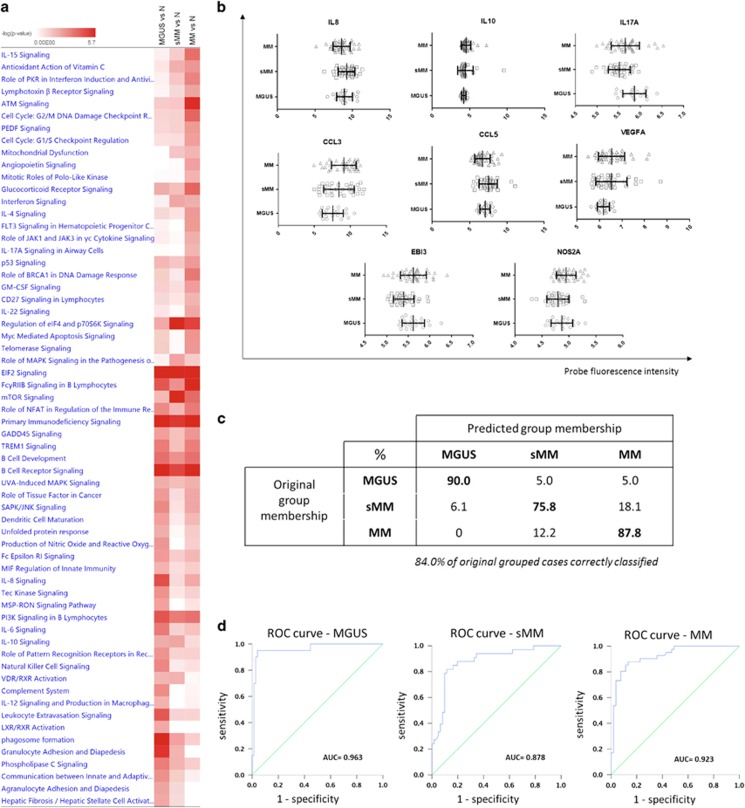
(**a**) Comparison analysis to investigate the main affected canonical pathway during evolution from MGUS to MM. Color intensity represents the degree of significance of pathway modulation in each disease condition. (**b**) Probe fluorescence intensity of the eight genes that resulted significantly associated with MGUS, sMM and MM condition after multinomial logistic regression analysis The range and interval of all axes was automatically determined to evidence differences in fluorescence distribution between different conditions. (**c**) Percentages of patients correctly classified according to the 8-genes model. (**d**) ROC curves built to evaluate the accuracy of the 8-genes model.

We compared GEP data of healthy donors, MGUS, sMM and MM patients from GSE47552 dataset to investigate the main canonical pathways modulated during disease progression. As shown in Figure 1a, IPA comparison between different disease conditions shows inflammatory and cytokine/chemokine signaling pathways as the most significantly modulated during the transition from MGSU to MM. This finding provided us the rational for selecting 20 candidate genes, known to be involved in B cells/PCs-mediated inflammation, and evaluating their relevance in predicting disease progression. The flow chart for the construction of the model is reported in Supplementary Figure 1. In univariate analysis, we investigated the 20 selected genes for their capability to correctly discriminate between the three disease states. We found 10 genes (IL15, IL17A, EBI3, CCL3, CCL5, LTB, CSF2, IFNG, RANKl and NOS2) with highly significant differential expression (*P*<0.01), 6 genes (IL2, IL8, IL10, TNF, TGFB1 and VEGFA) with significant differential expression (*P*<0.05 but >0.01) and 5 genes (IL6, IL12A, CCL2, LTA) not correlated with the disease status according to Kruskal–Wallis ANOVA test (Table 1). Subsequently, all significant variables underwent a multinomial logistic regression model (parameters and coefficients reported in Supplementary Table 3) that identified 8 genes (IL8, IL10, IL17A, CCL3, CCL5, VEGFA, EBI3 and NOS2) (Figure 1b) whose combination correctly assigned 84% of subjects to disease groups (Figure 1c). The model robustness was further confirmed by the high AUC (near 0.9) reported for the three ROC curves (Figure 1d).

### A 6-genes inflammatory score predicts survival of MM patients

**Figure 2:**
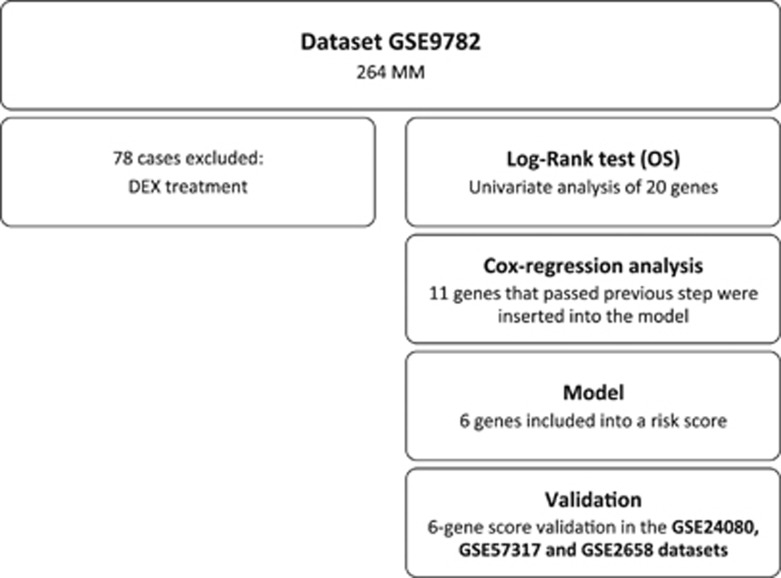
Algorithm for prognostic score identification.

**Figure 3:**
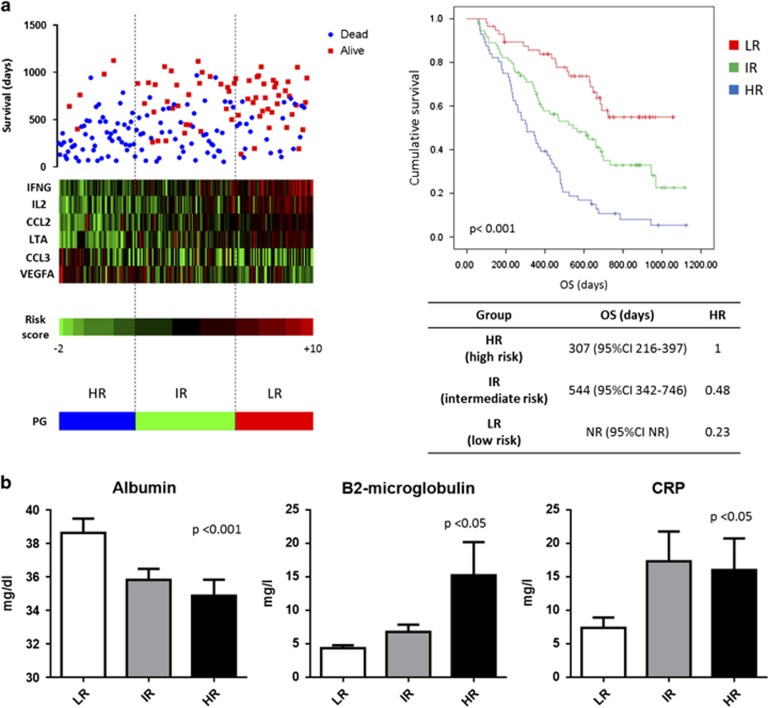
(**a**) On the left, heatmap reporting probe fluorescence intensity of six selected genes for each patient evaluated in accordance with its survival, prognostic score and PG. On the right, Kaplan–Meier curves reporting patients' survival according to their PG. Median survival and Hazard ratio values are reported below the curves. (**b**) Evaluation of correlation between PGs, the two variables forming the international staging system (albumin and B2-microglobulin) and CRP (c-reactive protein).

We further investigated whether selected inflammatory genes could be associated with MM patients' survival. To do that, we used as a training set for our model the GSE9782 dataset (flow chart is reported in Figure 2). We first performed a univariate survival analysis (log-rank test) to identify inflammation-related genes, whose expression was correlated with patients' survival, to select candidates for multivariate analysis. A total of 12 genes were identified and results are reported in Supplementary Table 4. We excluded IL6 from subsequent analysis due to excessive cross of survival curves. To evaluate if selected genes independently predicted patients' survival, we performed a multivariate regression analysis. Only 6 genes (IFNG, IL2, CCL2, CCL3, VEGF and LTA) showed independent predictive power (Supplementary Figure 2A). Of them, a higher expression of IFNG, IL2, CCL2 and LTA was associated with good prognosis, whereas a higher expression of CCL3 or VEGFA was associated with worse survival. Because of the fact that Cox-regression model reported a similar relative contribution (positive or negative) for each variable (data not shown) and to the need of building a model suitable for different gene expression platforms, we added a score to each gene (low expression=1, mid expression=2 and high expression=3) and then built a prognostic risk score (RS) as follows: IFNG+IL2+CCL2+LTA—VEGFA–CCL3. The RS may assume 13 different values and its capability to discriminate patients surviving more than 12, 18 or 24 months is shown in Supplementary Figure 2B. According to 33th and 66th percentile of the RS, patients were divided into three PGs: HR (high risk)=RS −2/+2; IR (intermediate risk)=RS 3/5; LR (low risk)=RS 6/10 (Figure 3a). The prognostic model was strongly associated with survival, with patients in LR group not reaching the median OS and experiencing a Hazard Ratio reduction of 77% (Figure 3a). Furthermore, a higher prognostic score (low risk) was significantly associated with high albumin and low B2-microglobulin levels, and consequently to a low ISS score. Interestingly, the LR group presented a low CRP value (Figure 3b).

### Validation of the inflammatory prognostic score in different patient datasets

**Figure 4:**
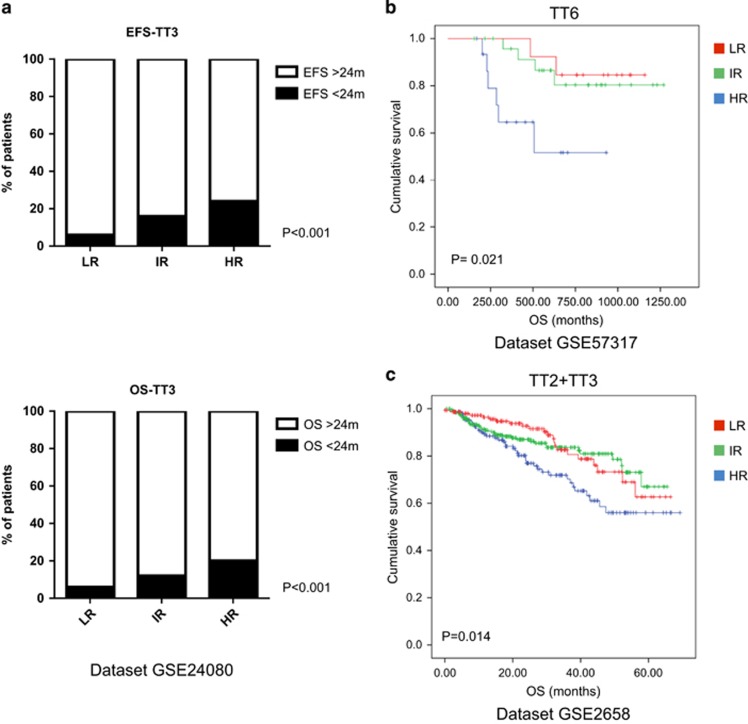
Validation of the PGs (based on the six variables prognostic score) in three different datasets: (**a**) The HR group included subjects with a higher risk to experience an event-free survival and OS lower than 24 months. (**b**, **c**) The HR group identified patients with the lowest OS.

To confirm the robustness of the inflammatory prognostic score, we validated our model in three independent datasets: GSE24080, GSE57317 and GSE2658. The GSE24080 only reported survival data in term of OS>24 or <24 months, anyway patients belonging to the LR group presented a significant higher number of long survival patients as compared to both IR and HR groups (Figure 4a; Supplementary Figure 3). Both GSE57317 and GSE2658 reported survival data, and in both datasets patients in the HR group presented a significant shorter survival thus validating our model (Figures 4b and c; Supplementary Figure 3).

## Discussion

In this study, we investigated the role of 20 inflammation-related genes in predicting disease evolution and MM patients' survival. Firstly, we analyzed the expression of these genes in a GEP microarray dataset from purified PCs of MGUS-sMM-MM patients. Through a multinomial logistic regression analysis, we identified an 8-genes signature able to discriminate with high precision the three different conditions. We found a consistent upregulation of CCL3, VEGFA and NOS2 in MM as compared with MGUS, which are known to attract myeloid cells such as neutrophils and monocytes at the inflammatory site.^30, 31^ Notably, we found a consistent decrease of LTA and LTB during disease evolution; these genes code for cytokines (TNF-β and TNF-C, respectively) essential for adaptive immune-response due to their role in follicular dendritic cell maturation, Th1 polarization and organization and activation of secondary as well as tertiary lymphoid organs.^23^ These findings led us to hypothesize that PCs progressively shift the BMM toward a pro-inflammatory and immunosuppressive shape, which drives disease evolution.

We then evaluated whether differential expression of selected inflammatory genes could predict patients survival. We identified IFNG, IL2, LTA and CCL2 as correlated with favorable prognosis, whereas CCL3 and VEGFA were associated with adverse outcome. On these bases, we built a prognostic risk score with patients classified into three PGs. We validated our prognostic score in three independent cohorts of patients with MM. Patients who experienced the longest survival presented high levels of IFNG, IL2, LTA and CCL2; the first three code for main cytokines driving Th1 response and T/NK-cell proliferation and cytotoxic activity.^32, 33^ Conversely, CCL2 has been described to have a role in both tumor progression and immune activation.^34^ Indeed, different studies reported its role in angiogenesis and MM homing to the BM as well as in the recruitment of tumor-promoting macrophages and anti-tumor cytotoxic γδ T lymphocytes.^34, 35^ It is conceivable that increased production of all these cytokines by MM cells make CCL2 predominantly tumor-suppressive, resulting in the promotion of Th1 response, which might lead to increased patients survival.^36^ On the other side, we identified VEGFA and CCL3 as overexpressed in patients with adverse outcome. VEGFA, which is secreted by MM cells and components of the BMM, promotes neo-angiogenesis, MM cells survival, migration, and has an immune-suppressive role.^37^ CCL3 is produced mainly by MM cells and acts as chemoattractant for monocyte-derived cells which, within the BMM, differentiate into macrophages or osteoclasts. These cells in turn promote inflammation, angiogenesis, osteolytic lesions and immune-response impairment.^38^ Both cytokines highlight the inflammatory microenvironment and are involved in MM-associated bone disease, worsening patients' prognosis.^39, 40^

Inflammation is an hallmark of cancer development.^41^ Indeed, different studies have already demonstrated a strong correlation between chronic inflammation and increased risk of cancer. Moreover, the chemopreventive role of aspirin and other NSAIDs has been clearly demonstrated.^42^ Along this line, recent clinical trials revealed a promising therapeutic activity of anti-inflammatory compounds such as aspirin and curcumin in both MGUS and sMM patients.^43, 44^

Furthermore, inflammation could also reduce the activity of current anti-cancer treatment (both cytotoxic and immunotherapies), by impairing effective immune-response against tumor cells.^12, 45, 46, 47, 48^ Accordingly, there is a growing body of evidence underlining that an immunogenic response following anti-cancer therapies produce long-lasting responses.^46, 49, 50, 51, 52^ This event implies immunogenic cell death (ICD), which is characterized by generation of an immune-activating microenvironment where dying cells (i) are recognized by professional antigen-presenting cells (APCs) due to the surface expression of several ‘eat-me' molecules such as calreticulin and/or HSP70/90; and (ii) attract and activate APCs to promote an efficient anti-tumor Th1/γδ T/CD8 cell response, by releasing ATP, HMGB1 and type 1 IFNs.^53^ On these bases, we believe that a BMM where MM cells secrete Th1 cytokines (IFNG, IL2, LTA), attract γδ T cells (through CCL2 production) and produce low amount of inflammatory/immunosuppressive cytokines (VEGF and CCL3),^54, 55^ represents the best condition for therapeutic activity of ICD-inducers bortezomib^56^ or doxorubicin,^57^ and of the immunomodulatory drugs thalidomide, lenalidomide and pomalidomide.^9^

Although our results clearly underscore the role of several inflammatory genes in MM pathogenesis, we underline several limitations of our study. First of all, our analysis is performed on candidate genes. This may however represent a strength, due to the fact that genes are selected on the basis of well-defined pathways, and a weakness, due to the fact that information related to uninvestigated genes is unavoidably lost. Nevertheless, we demonstrated that inflammatory pathways are indeed among the most modulated during disease evolution and we validated our prognostic score in three different annotated datasets. In addition, we cannot exclude the influence of the small percentage of non-myeloma cells on the results of our analysis. Indeed, we tried to reduce this bias by working on genes that code for molecules produced by PCs or B cells. Further limitations are that our model is trained and validated on microarray based platform, and that the disease evolution model still need to be validated. Indeed, our findings should be considered ‘hypothesis-generating' and future prospective validation of the models must rely on different technologies, such as qRT-PCR and/or RNAseq.

Nonetheless, with our research, we identified potential targets, which might be of major relevance for antagonizing the disease evolution or for the treatment of symptomatic-MM patients. It is important to underscore that the simultaneous targeting of these different pathways rather than the single molecules inhibition might effectively reduce inflammation and induce Th1/γδ/CD8 response, finally resulting in increased patients' survival. To this aim, current research will gain the best advantage from the new emerging scenario of functional network of non-coding RNAs, such as miRNAs and long non-coding RNAs, that already demonstrated their role in the control of different (immune-) cell functions and in cancer biology, and are approaching the clinical side.^58, 59, 60, 61^ In conclusion, we provide proof-of-concept that MM cells drive a pro-inflammatory effect in the BMM, which is relevant in the disease evolution. The inflammatory signatures here reported, which differentiate disease phases and offer a novel prognostic tool, might be relevant for the design of novel individualized treatment of MM.

## Figures and Tables

**Table 1:** Univariate association of inflammatory genes with disease conditions

*Gene*	*Condition*	*Mean intensity*	P*-value* *MW test*	P*-value* *KW test*
*IL2*	MGUS	2.59		**0.02**
	sMM	2.62	0.31	
	MM	2.67	**0.01**	
*IL6*	MGUS	5.02		0.42
	sMM	5.30	0.50	
	MM	5.56	0.20	
*IL8*	MGUS	8.94		**0.03**
	sMM	9.22	0.33	
	MM	8.56	0.15	
*IL10*	MGUS	4.21		**0.03**
	sMM	4.43	0.74	
	MM	4.54	**0.02**	
*IL12A*	MGUS	5.56		0.07
	sMM	5.96	**0.05**	
	MM	5.93	**0.03**	
*IL15*	MGUS	3.98		**<0.01**
	sMM	4.32	**<0.01**	
	MM	4.38	**<0.01**	
*IL17A*	MGUS	5.86		
	sMM	5.51	**<0.01**	**<0.01**
	MM	5.65	**<0.01**	
*EBI3*	MGUS	5.62		
	sMM	5.40	**<0.01**	**<0.01**
	MM	5.62	0.71	
*CCL2*	MGUS	6.20		0.10
	sMM	5.93	0.18	
	MM	6.41	0.64	
*CCL3*	MGUS	7.55		**<0.01**
	sMM	8.41	0.17	
	MM	9.10	**<0.01**	
*CCL5*	MGUS	7.06		**<0.01**
	sMM	7.49	0.20	
	MM	6.71	0.06	
*LTA*	MGUS	5.122		0.11
	sMM	5.044	0.19	
	MM	4.984	**0.04**	
*LTB*	MGUS	8.05		**<0.01**
	sMM	7.79	**<0.01**	
	MM	7.83	**<0.01**	
*CSF2*	MGUS	3.26		**<0.01**
	sMM	3.19	0.13	
	MM	3.34	0.13	
*TNFA*	MGUS	5.34		**0.05**
	sMM	5.42	0.75	
	MM	5.55	0.06	
*IFNG*	MGUS	3.57		**<0.01**
	sMM	4.16	**<0.01**	
	MM	3.60	0.51	
*TGFB1*	MGUS	7.62		**0.05**
	sMM	8.01	**0.01**	
	MM	7.99	**0.04**	
*RANKL*	MGUS	3.71		**0.01**
	sMM	3.59	**0.02**	
	MM	3.71	0.57	
*VEGFA*	MGUS	6.18		**0.04**
	sMM	6.53	0.07	
	MM	6.54	**0.01**	
*NOS2*	MGUS	4.87		**<0.01**
	sMM	4.80	0.22	
	MM	4.95	0.15	

Abbreviations: MGUS, monoclonal gammopathy of undetermined significance; MM, multiple myeloma; sMM, smoldering MM.

This table include the results of Mann–Whitney (MW) and Kruskall–Wallis (KW) tests in which each of the 20 candidates genes were evaluated for their association with each disease condition. Values in bold are statistically significant.
