# Supplementary material for: A gene expression inflammatory signature specifically predicts multiple myeloma evolution and patients survival
Source: Blood Cancer J. 2016 Dec 16;6(12):e511–. doi: 10.1038/bcj.2016.118 (PMC5223153; doi:10.1038/bcj.2016.118)
Supplement: Supplementary Table 1 [file bcj2016118x1.docx]

| Dataset | Samples | Platform | Patients’ status | Description of the study |
| --- | --- | --- | --- | --- |
| GSE47552 | 99 | Affymetrix Human Gene 1.0 ST Array | Chemo-naïve | Transcriptome profiling of CD138+ immunomagnetically selected plasma cells from 20 MGUS, 33 sMM, 41 MM and 5 healthy donors |
| GSE9782 | 264 | Affymetrix Human Genome U133A/B Array | Pretreated | Expression profiling of purified and enriched myeloma samples collected from 186 patients treated with bortezomib and 78 treated with dexamethasone in phase 2 and phase 3 clinical trials of bortezomib (APEX, M34101-040, SUMMIT and CREST trials) |
| GSE24080 | 559 | Affymetrix Human Genome U133 Plus 2.0 Array | Chemo-naïve | Expression profiling of plasma cells enriched by anti-CD138 immunomagnetic bead selection extracted from bone marrow aspirates of 340 patients enrolled on total therapy 2 and 214 patients enrolled on total therapy 3 |
| GSE57317 | 55 | Affymetrix Human Genome U133 Plus 2.0 Array | Pretreated | Expression profiling of CD138+ selected cells from bone marrow of 55 MM patients before treatment with total teraphy 6 |
| GSE2658 | 559 | Affymetrix Human Genome U133 Plus 2.0 Array | Chemo-naïve | Expression profiling of CD138+ selected plasma cells from pre-treatment bone marrow aspirates from 351 MM patients treated with total therapy 2 and 208 patients treated with total therapy 3 |

**Supplementary table 1**
