# Supplementary material for: A gene expression inflammatory signature specifically predicts multiple myeloma evolution and patients survival
Source: Blood Cancer J. 2016 Dec 16;6(12):e511–. doi: 10.1038/bcj.2016.118 (PMC5223153; doi:10.1038/bcj.2016.118)
Supplement: Supplementary Table 2 [file bcj2016118x2.docx]

| GENE | Rationale | reference |
| --- | --- | --- |
| IL2 | Inflammatory cytokine produced by B cells to support memory T cells generation | **23** |
| IL6 | Inflammatory cytokine produced by B cells to support Th1, Th17 and follicular Th (Thf) response | **23, 28** |
| IL8 | Inflammatory cytokine produced by B cells | **22** |
| IL10 | Immuno-regulatory cytokine produced by regulatory B cells | **22, 23, 28** |
| IL12A | Cytokine expressed by B cells | **23** |
| IL15 | Inflammatory cytokine produced by B cells to support CD8 T cell response | **27** |
| IL17A | Inflammatory cytokine produced by  - B cells to support the accumulation of IL10 producing neutrophils thus reducing Th1 response  - MM cells to support their own growth and bone disease | **23**  **26** |
| EBI3 | Immuno-regulatory cytokine (IL35) produced by  - normal plasma cells in different auto-immune diseases  - regulatory B cells to reduce Th1 and Th17 response | **23**  **25** |
| CCL2 | Inflammatory cytokine produced by MM cells involved in MM migration | **21** |
| CCL3 | Inflammatory cytokine produced by  - B cells to regulate Th1 response  - MM cells to induce bone disease | **23**  **29** |
| CCL5 | Cytokine produced by B cells in response to inflammatory stimuli | **25** |
| LTA | Inflammatory cytokine produced by B cells involved in T helper lymphocytes polarization, expansion of lymph node, follicular dendritic cells activation, marginal zone development | **23** |
| LTB | Inflammatory cytokine produced by B cells involved in T helper lymphocytes polarization, expansion of lymph node, follicular dendritic cells activation, marginal zone development | **23** |
| CSF2 | Inflammatory cytokine produced by B cells to induce IL12 secretion by DCs | **23** |
| TNFA | Inflammatory cytokine produced by:  - MM cells involved in MM migration  - B cells involved in Th1 response | **21**  **23, 28** |
| IFNG | Inflammatory cytokine produced by B cells to support Th1 response and macrophage activation | **23** |
| TGFB1 | Immuno-regulatory cytokine produced by B cells | **22** |
| RANKL | Inflammatory cytokine produced by MM cells involved in MM associated bone disease | **19** |
| VEGFA | Cytokine produced by B cells in presence of an inflammatory microenvironment | **24** |
| NOS2 | Expressed by MM cells and responsible for the synthesis of nitric oxide and for the response to IL6 stimulation | **20** |

**Supplementary table 2**
