# Supplementary material for: A gene expression inflammatory signature specifically predicts multiple myeloma evolution and patients survival
Source: Blood Cancer J. 2016 Dec 16;6(12):e511–. doi: 10.1038/bcj.2016.118 (PMC5223153; doi:10.1038/bcj.2016.118)
Supplement: Supplementary Table 3 [file bcj2016118x3.docx]

| **Supplementary table 3** | | | | | | | | | |
| --- | --- | --- | --- | --- | --- | --- | --- | --- | --- |
| group^a^ | | B | Std. Error | Wald | df | Sig. | Exp(B) | 95% Confidence Interval for Exp(B) | |
|  |  |  |  |  |  |  |  | Lower Bound | Upper Bound |
| sMM | Intercept | 6.610 | 3.653 | 3.274 | 1 | .070 |  |  |  |
|  | [NIL10=1] | 1.889 | 1.439 | 1.725 | 1 | .189 | 6.616 | .394 | 110.989 |
|  | [NIL10=2] | -.519 | 1.493 | .121 | 1 | .728 | .595 | .032 | 11.100 |
|  | [NIL10=3] | 0^b^ | . | . | 0 | . | . | . | . |
|  | [NIL17a=1] | 7.395 | 2.747 | 7.246 | 1 | .007 | 1627.013 | 7.468 | 354479.956 |
|  | [NIL17a=2] | 6.347 | 2.271 | 7.811 | 1 | .005 | 570.569 | 6.658 | 48898.702 |
|  | [NIL17a=3] | 0^b^ | . | . | 0 | . | . | . | . |
|  | [NNOS2A=1] | -5.970 | 2.758 | 4.684 | 1 | .030 | .003 | 1.147E-005 | .569 |
|  | [NNOS2A=2] | -3.324 | 2.236 | 2.210 | 1 | .137 | .036 | .000 | 2.883 |
|  | [NNOS2A=3] | 0^b^ | . | . | 0 | . | . | . | . |
|  | [NCCL3=1] | -8.313 | 3.447 | 5.816 | 1 | .016 | .000 | 2.854E-007 | .211 |
|  | [NCCL3=2] | -5.896 | 2.923 | 4.069 | 1 | .044 | .003 | 8.935E-006 | .846 |
|  | [NCCL3=3] | 0^b^ | . | . | 0 | . | . | . | . |
|  | [NVEGFA=1] | -1.986 | 1.488 | 1.782 | 1 | .182 | .137 | .007 | 2.534 |
|  | [NVEGFA=2] | -2.738 | 1.523 | 3.232 | 1 | .072 | .065 | .003 | 1.280 |
|  | [NVEGFA=3] | 0^b^ | . | . | 0 | . | . | . | . |
|  | [NEBI3=1] | 3.775 | 1.795 | 4.424 | 1 | .035 | 43.600 | 1.293 | 1469.645 |
|  | [NEBI3=2] | .710 | 1.507 | .222 | 1 | .638 | 2.034 | .106 | 38.987 |
|  | [NEBI3=3] | 0^b^ | . | . | 0 | . | . | . | . |
|  | [NIL8=1] | -.228 | 1.437 | .025 | 1 | .874 | .796 | .048 | 13.301 |
|  | [NIL8=2] | -1.583 | 1.163 | 1.852 | 1 | .174 | .205 | .021 | 2.007 |
|  | [NIL8=3] | 0^b^ | . | . | 0 | . | . | . | . |
|  | [NCCL5=1] | 2.230 | 1.873 | 1.418 | 1 | .234 | 9.301 | .237 | 365.218 |
|  | [NCCL5=2] | -.130 | 1.130 | .013 | 1 | .908 | .878 | .096 | 8.044 |
|  | [NCCL5=3] | 0^b^ | . | . | 0 | . | . | . | . |
| MM | Intercept | 7.823 | 3.603 | 4.714 | 1 | .030 |  |  |  |
|  | [NIL10=1] | .624 | 1.453 | .184 | 1 | .668 | 1.865 | .108 | 32.185 |
|  | [NIL10=2] | -2.359 | 1.503 | 2.465 | 1 | .116 | .095 | .005 | 1.797 |
|  | [NIL10=3] | 0^b^ | . | . | 0 | . | . | . | . |
|  | [NIL17a=1] | 9.512 | 2.845 | 11.177 | 1 | .001 | 13523.612 | 51.189 | 3572787.846 |
|  | [NIL17a=2] | 7.390 | 2.328 | 10.081 | 1 | .001 | 1620.022 | 16.914 | 155164.871 |
|  | [NIL17a=3] | 0^b^ | . | . | 0 | . | . | . | . |
|  | [NNOS2A=1] | -6.763 | 2.808 | 5.799 | 1 | .016 | .001 | 4.702E-006 | .284 |
|  | [NNOS2A=2] | -3.221 | 2.231 | 2.085 | 1 | .149 | .040 | .001 | 3.162 |
|  | [NNOS2A=3] | 0^b^ | . | . | 0 | . | . | . | . |
|  | [NCCL3=1] | -10.620 | 3.504 | 9.183 | 1 | .002 | 2.443E-005 | 2.541E-008 | .023 |
|  | [NCCL3=2] | -7.620 | 2.967 | 6.597 | 1 | .010 | .000 | 1.464E-006 | .164 |
|  | [NCCL3=3] | 0^b^ | . | . | 0 | . | . | . | . |
|  | [NVEGFA=1] | -3.749 | 1.563 | 5.754 | 1 | .016 | .024 | .001 | .504 |
|  | [NVEGFA=2] | -2.928 | 1.553 | 3.555 | 1 | .059 | .054 | .003 | 1.122 |
|  | [NVEGFA=3] | 0^b^ | . | . | 0 | . | . | . | . |
|  | [NEBI3=1] | .781 | 1.929 | .164 | 1 | .685 | 2.184 | .050 | 95.694 |
|  | [NEBI3=2] | -1.532 | 1.611 | .904 | 1 | .342 | .216 | .009 | 5.085 |
|  | [NEBI3=3] | 0^b^ | . | . | 0 | . | . | . | . |
|  | [NIL8=1] | 2.213 | 1.542 | 2.059 | 1 | .151 | 9.144 | .445 | 187.879 |
|  | [NIL8=2] | -.668 | 1.301 | .263 | 1 | .608 | .513 | .040 | 6.570 |
|  | [NIL8=3] | 0^b^ | . | . | 0 | . | . | . | . |
|  | [NCCL5=1] | 6.058 | 2.030 | 8.905 | 1 | .003 | 427.646 | 7.999 | 22862.591 |
|  | [NCCL5=2] | 1.712 | 1.359 | 1.589 | 1 | .208 | 5.542 | .387 | 79.461 |
|  | [NCCL5=3] | 0^b^ | . | . | 0 | . | . | . | . |
| a. The reference category is: MGUS. | | | | | | | | | |
| b. This parameter is set to zero because it is redundant. | | | | | | | | | |
