# Supplementary material for: A gene expression inflammatory signature specifically predicts multiple myeloma evolution and patients survival
Source: Blood Cancer J. 2016 Dec 16;6(12):e511–. doi: 10.1038/bcj.2016.118 (PMC5223153; doi:10.1038/bcj.2016.118)
Supplement: Supplementary Table 4 [file bcj2016118x4.docx]

| GENE | Global Log-Rank test p value |
| --- | --- |
| IL2 | **<0.01** |
| IL6 | **<0.01** |
| IL8 | 0.38 |
| IL10 | **0.06** |
| IL12A | 0.25 |
| IL15 | 0.37 |
| IL17A | 0.10 |
| EBI3 | **0.05** |
| CCL2 | **<0.01** |
| CCL3 | **0.07** |
| CCL5 | 0.29 |
| LTA | **<0.01** |
| LTB | 0.89 |
| CSF2 | **0.06** |
| TNFA | 0.564 |
| IFNG | **<0.01** |
| TGFB1 | 0.27 |
| RANKL | **<0.01** |
| VEGFA | **0.07** |
| NOS2 | **<0.01** |

**Supplementary table 4**
