# Supplementary material for: A gene expression inflammatory signature specifically predicts multiple myeloma evolution and patients survival
Source: Blood Cancer J. 2016 Dec 16;6(12):e511–. doi: 10.1038/bcj.2016.118 (PMC5223153; doi:10.1038/bcj.2016.118)
Supplement: Supplementary Figure Legends [file bcj2016118x5.docx]

**Supplementary figure legends**

**Supplementary figure 1**

Algorithm for the identification of inflammatory gene signature associated with disease evolution.

**Supplementary figure 2**

**A:** Kaplan Meier curves of the 6 genes strongly associated with patients’ overall survival (OS) according to multivariate Cox regression analysis.

**B:** Receiving operating characteristic (ROC) curves evaluating the performance of the inflammatory risk score in predicting patients’ with an overall survival (OS) longer than 12, 18 or 24 months.

**Supplementary figure 3**

Validation of inflammatory risk score in all the sub-groups of dataset GSE24080 and GSE2658
