# Supplementary figures and images for: A gene expression inflammatory signature specifically predicts multiple myeloma evolution and patients survival
Source: Blood Cancer J. 2016 Dec 16;6(12):e511–. doi: 10.1038/bcj.2016.118 (PMC5223153; doi:10.1038/bcj.2016.118)

## Supplementary figure 1

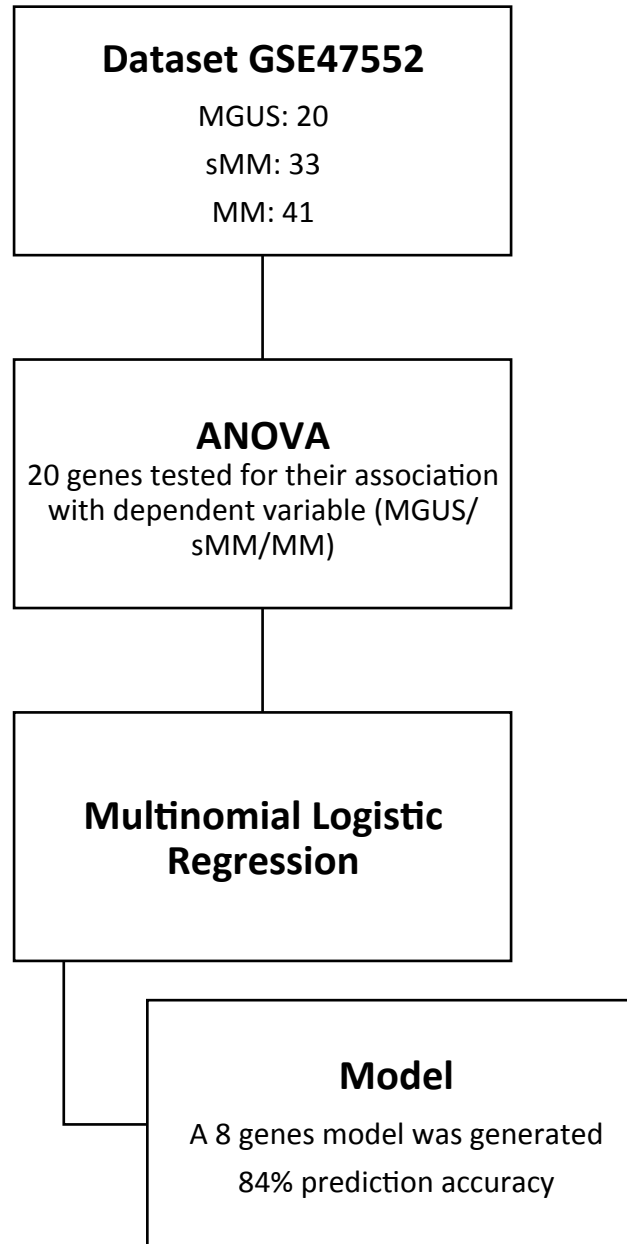

Supplement: Supplementary Figure 1 [file bcj2016118x6.pdf]

Supplementary figure 2

A

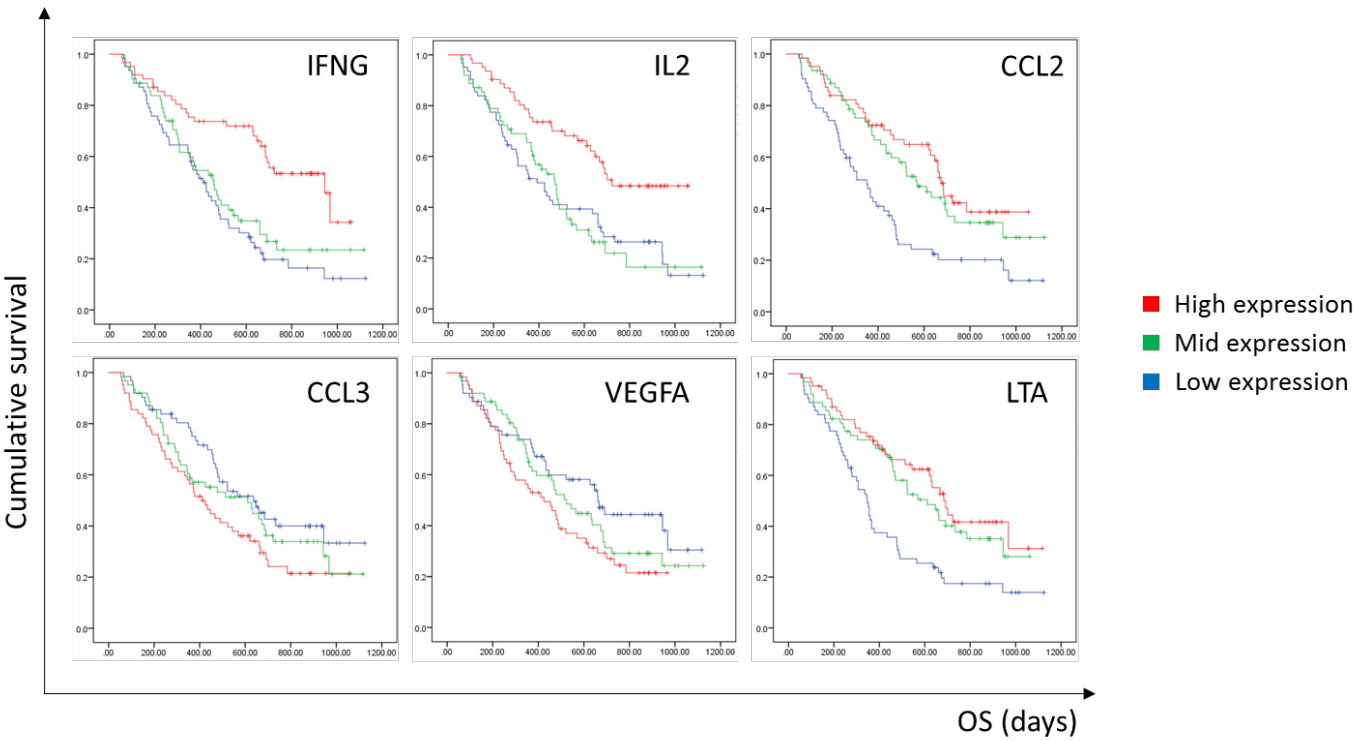

B

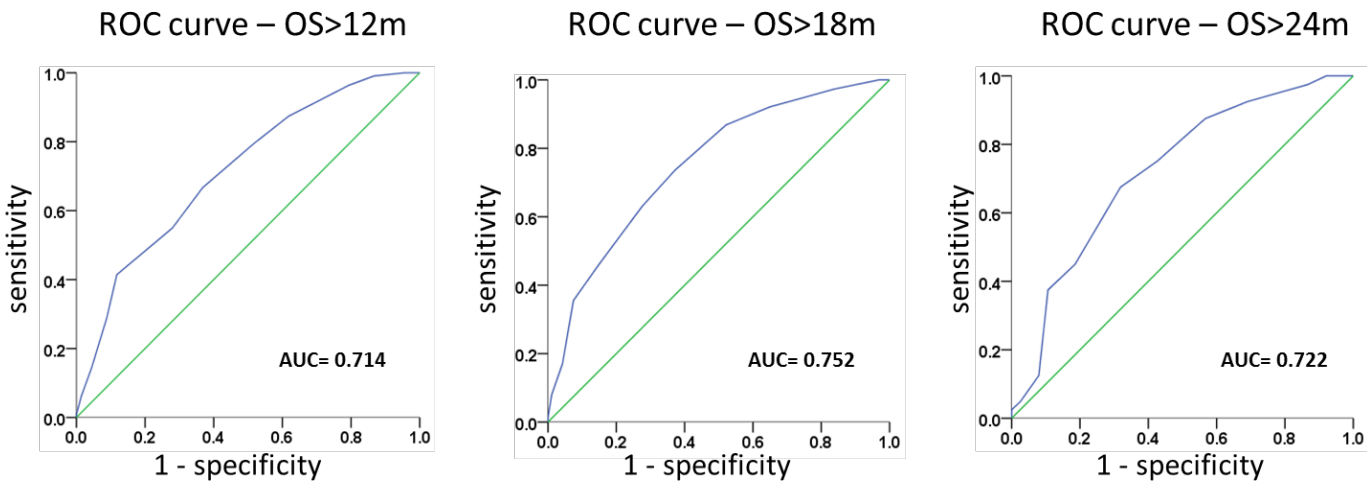

Supplement: Supplementary Figure 2 [file bcj2016118x7.pdf]

# Supplementary figure 3

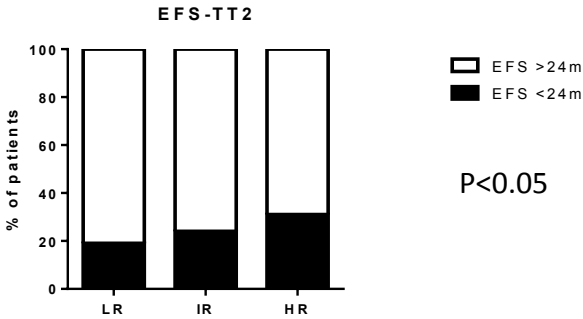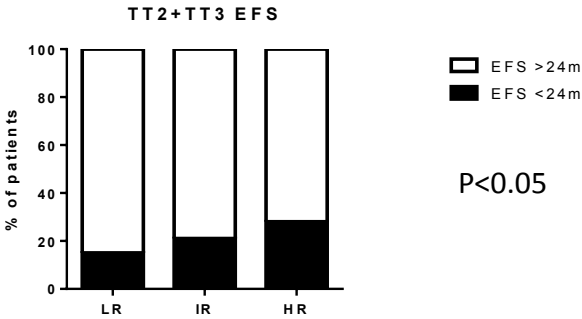

Dataset GSE24080

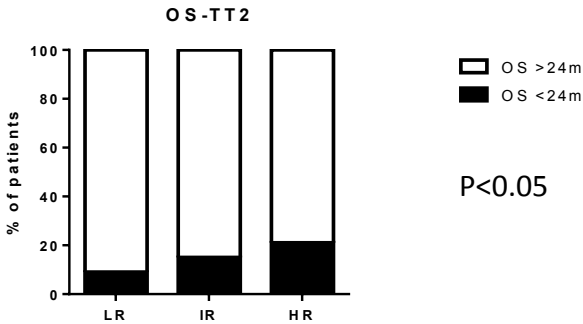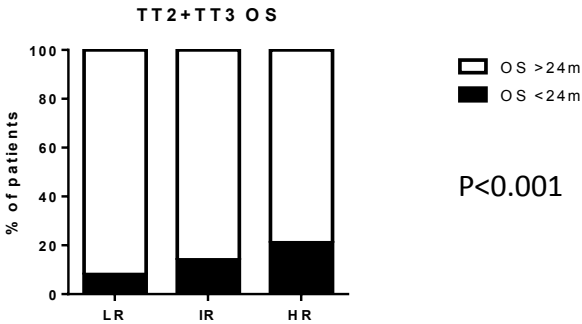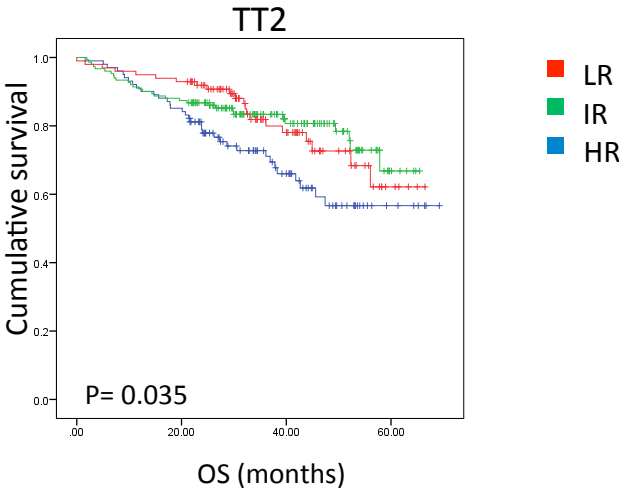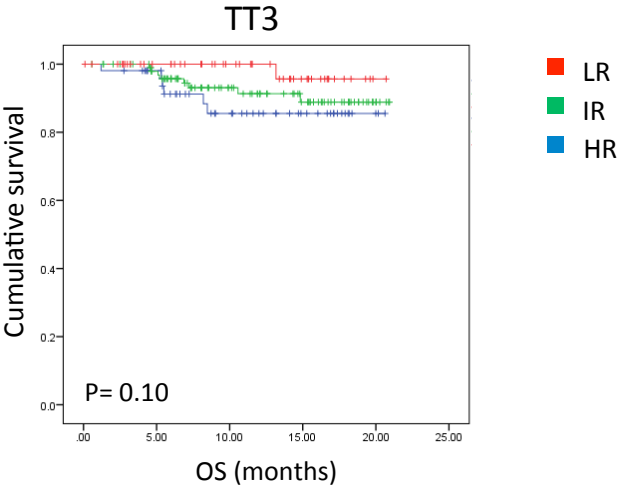

Dataset GSE2658

Supplement: Supplementary Figure 3 [file bcj2016118x8.pdf]
